# Identification of 10 SUMOylation-Related Genes From Yellow Catfish *Pelteobagrus fulvidraco*, and Their Transcriptional Responses to Carbohydrate Addition *in vivo* and *in vitro*

**DOI:** 10.3389/fphys.2018.01544

**Published:** 2018-11-08

**Authors:** Shui-Bo Yang, Xiao-Ying Tan, Dian-Guang Zhang, Jie Cheng, Zhi Luo

**Affiliations:** ^1^Key Laboratory of Freshwater Animal Breeding, Ministry of Agriculture, Fishery College, Huazhong Agricultural University, Wuhan, China; ^2^Collaborative Innovation Center for Efficient and Health Production of Fisheries in Hunan Province, Changde, China

**Keywords:** SUMOylation, molecular characterization, transcriptional responses, carbohydrate, fish

## Abstract

SUMOylation is a kind of important post-translational modification. In the present study, we identified 10 key genes involved in SUMOylation and deSUMOylation (*sumo1, sumo2, sumo3, sae1, uba2, ubc9, pias1, senp1, senp2*, and *senp3*) in yellow catfish *Pelteobagrus fulvidraco*, investigated their tissue expression patterns and transcriptional responses to carbohydrate addition both *in vivo* and *in vitro*. All of these members shared similar domains to their orthologous genes of other vertebrates. Their mRNAs were widely expressed in all the tested tissues, but at variable levels. Dietary carbohydrate levels differentially influenced the mRNA levels of these genes in liver, muscle, testis, and ovary of yellow catfish. Their mRNA levels in primary hepatocytes were differentially responsive to glucose addition. Our study would contribute to our understanding into the molecular basis of SUMOylation modification and into the potential SUMOylation function in the carbohydrate utilization in fish.

## Introduction

SUMOylation is a kind of important post-translational modification. It is characterized by SUMO proteins which covalently binds to the ε-amino group of lysine residues in intracellular target proteins in an ATP-dependent process (Gareau and Lima, 2010). To date, four SUMO proteins have been identified and characterized in humans: *SUMO 1, SUMO 2, SUMO 3*, and *SUMO 4* (Pseudogene) (Krumova and Weishaupt, 2013). During the SUMOylation modification, a series of enzymatic reactions mediate the process. These enzymes include the heteromeric activating enzyme E1 (Uba2), the conjugation enzyme E2 (Ubc9), and SUMO E3 ligases represented by PIAS family (Johnson, 2004; Hay, 2005). SUMOylation plays a central role in cell homeostasis by regulating a large number of key biological processes such as signal transduction, nucleoplasm transport, transcription regulation, DNA repair, and cell cycle (Johnson, 2004; Hay, 2005; Yang and Paschen, 2009). In addition, SUMOylation is a dynamic process which can be reversed by a family of Sentrin/SUMO-specific protease (Bailey and O’Hare, 2004; Hay, 2005). SENPs act to deSUMOylate the proteins by cleaving the covalent conjugation between SUMO and its target (Wilkinson and Henley, 2010).

To date, most studies involved in SUMOylation and deSUMOylation mainly focuse on mammals. The data is still very scarce in aquatic organisms, including fish. To our best knowledge, only partial genes in the process of SUMOylation and deSUMOylation have been identified in very limited fish, such as in grouper [*sumo1* and *sumo2* (Xu et al., 2016)], medaka fish [*senp1* (Obata et al., 2013)], zebrafish [*ubc9* (Yuan et al., 2010)], channel catfish [*ubc9* (Chen et al., 2010)], yellow croaker [*ubc9* (Zhou et al., 2009)], half-smooth tongue sole [*ubc9* (Hu and Chen, 2013)], grass carp [*pias1* (Wong, 2013)], and rainbow trout [*sumo1, sumo2, sumo3, sae1, sae2, ubc9*, and *pias1* (Zang, 2013)]. Very limited studies also explored their mRNA tissue expression profiles of genes involving SUMOylation and deSUMOylation processes (Hu and Chen, 2013; Xu et al., 2016).

SUMOylation modification of proteins plays important roles in the function, compartmentalization and stability of target proteins, contributing to the regulation of diverse processes (Bailey and O’Hare, 2004). The exploration of the emerging roles of SUMOylation modification in nutrient metabolism is a growing and fascinating field. In mammals, Huang et al. (2013) suggested that SUMOylation mediated the high carbohydrate (glucose)-induced process of NF-κB signaling activation. Generally speaking, compared to mammals, fish show relatively low capacity of utilization for dietary carbohydrate. However, compared to dietary lipid and proteins, carbohydrates are relatively inexpensive and a readily available source of energy, which makes their inclusion in the diet attractive. Previously, Ye et al. (2009) found that carbohydrate could cause protein-sparing effect in the diets for juvenile yellow catfish *Pelteobagrus fulvidraco*, a widely cultured freshwater teleost in China and other Asian countries. Since SUMOylation and deSUMOylation mediate the regulation of many transcriptional factors involved in nutrient metabolism, we are eager to know the effects of dietary carbohydrate levels on transcriptional responses of these genes. To this end, the present study identified the cDNA sequences of 10 key genes in SUMO- and deSUMOylation (*sumo1, sumo2, sumo3, sae1, uba2, ubc9, pias1, senp1, senp2*, and *senp3*) in yellow catfish, and examine their transcriptional responses in several tissues of yellow catfish fed different levels of dietary carbohydrate. Taken together, this study provided the basis for further exploring the biological characteristics of SUMO- and deSUMOylation and their potential functions for the utilization of dietary carbohydrate.

## Materials and Methods

The experiment is divided into three parts. In Experiment 1, we cloned sumoylation-related genes and explored their tissue distribution of gene expression. Experiment 2 was conducted to determinate their transcriptional responses to different levels of dietary carbohydrate. Experiment 3 was taken to determine the effect of glucose incubation on mRNA expression of these genes in primary hepatocytes from yellow catfish. The experimental protocol was approved by the Committee of Huazhong Agricultural University on the Ethics of Laboratory Animal Experiments.

### Experiment 1: Cloning and Tissue Distribution of Gene Expression

#### RNA Isolation and cDNA Cloning

The experimental protocols were similar to those described in the study by Wei et al. (2017). Yellow catfish (23.5 ± 3.3 g, mean ± SEM) were obtained from a local commercial farm. After 2-week acclimation, fish were fasted for 24 h and then euthanized with MS-222 (100 mg/L). Heart, liver, muscle, brain, kidney, intestine, spleen, mesenteric fat, testis, gill, and ovary were removed and stored at -80°C for subsequent tissue expression analysis. Total RNA was extracted using TRIzol RNA reagent (Invitrogen, United States) based on the acid guanidinium thiocyanate–phenol–chloroform extraction method. The quality of total RNA was check by agarose gel electrophoresis, and determined by Nanodrop ND-2000 spectrophotometer (1.8 < OD_260_/OD_280_ < 2.0, OD_260_/OD_230_ > 1.5). The nested 3′ and 5′ RACE PCR were performed to obtain the 3′ and 5′ end sequences with a SMART RACE cDNA Amplification Kit (Clontech, United States). The primers were shown in Supplementary Table 1.

#### Sequence Analysis

The full-length cDNA sequences of *sumo1-3, sae1/uba2, ubc9, pias1, senp2/senp3*, and partial sequences of *senp1* were edited and analyzed using the program EDITSEQ (DNAstar) to search for the open reading frame (ORF). They were then translated into amino acid sequences using standard genetic codes. Sequence alignments and percentage of amino acid conservation were assessed by Clustal-W multiple alignment algorithm. Domains were analyzed with the online CDD tool at NCBI^1^ and the SMART program^2^. For phylogenetic analysis, multiple sequence alignments were made with MAFFT using an amino acid model at the GUIDANCE web-server^3^, which pruned unreliably aligned regions by rejecting columns with confidence scores below 0.93. The phylogenetic trees were generated through neighbor-joining (NJ) method with MEGA 6.0 (Tamura et al., 2013) based on the JTT + G model (Jones et al., 1992), and the best-fit model of sequence evolution was obtained by ML model selection. Bootstrap sampling was reiterated 1000 times.

### Experiment 2: Effects of Dietary Carbohydrate Levels on mRNA Expression of Genes

Three experimental diets were formulated with dietary carbohydrate levels at 17.2% (low), 22.8% (moderate), and 30.2% (high), based on our published studies (Ye et al., 2009). Starch was used as dietary carbohydrate sources. Yellow catfish were obtained from a local fish farm (Wuhan, China). After 2-week acclimation, 270 uniformly size fish (4.1 ± 0.01 g, mean ± SEM) were randomly stocked to 9 circular fiberglass tanks, 30 fish for each tank. Each diet was assigned to three tanks in a completely randomized manner. The feeding protocols were similar to those in our recent study (Wei et al., 2017, 2018). The experiment lasted for 10 weeks. During the experiment, water temperature ranged from 25.7 to 28.6°C. Dissolved oxygen and NH4-N were 5.87–6.41 and 0.107–0.142 mg/L, respectively.

#### Sampling

At the end of the experiment, all fish were fasted for 24 h. Fish were killed (MS222 at a dose of 100 mg/L). Then, fish were dissected on ice to obtain liver, muscle, testis, and ovary. All samples were quickly frozen in liquid nitrogen, and reserved at -80°C for the subsequent real-time quantitative PCR (qPCR) analysis.

#### Quantitative PCR

Quantitative PCR (qPCR) was carried out according to the methods described in our previous studies (Cheng et al., 2017; Wei et al., 2017, 2018). The primer sequences of each gene used in this analysis are given in Supplementary Table 2. A set of 10 housekeeping genes (*18S* rRNA, *β-actin, rpl7, tuba, b2m, elfa, gapdh, tbp, hprt*, and *ubce*) were selected in order to test their transcriptional stability. The relative expression of genes was calculated by 2^-ΔΔ*Ct*^ method and GeNorm was used to normalize the geometric mean of two best combination genes under different experimental conditions.

### Experiment 3: Effect of Glucose Incubation on mRNA Expression of Genes in Primary Hepatocytes From Yellow Catfish

#### Primary Hepatocyte Isolation and Glucose Incubation

Primary hepatocytes were isolated from yellow catfish and cultured according to the methods described in our previous studies (Zhuo et al., 2014; Wei et al., 2018). Three glucose concentration, control (5.41 mmol/L glucose), 10 mM (9.75 mmol/L glucose), and 20 mM (20.38 mmol/L glucose), were used to incubate the isolated hepatocytes, respectively. Hepatocyte cell suspension (CS) was plated onto 25 cm^2^ flasks at the density of 1 × 10^6^ cells per mL, and three different levels of glucose were added and incubated at 28°C in humidified air containing 5% CO_2_. Sampling occurred at 12, 24, and 48 h, respectively. Each treatment was performed in triplicate.

#### Cell Livability Assay

The MTT assay was used to test the cell viability according to Zhuo et al. (2014). The wells containing the medium without any cells were used as the positive control. The results are presented as percentage cell viability, which was calculated as the ratio of absorbance A in the experimental well to A in the positive control well.

#### mRNA Preparation and qPCR Analysis

Hepatocytes were collected from 25 cm^2^ flasks for the total RNAs extracted by TRIzol regent (TaKaRa, Japan). Reverse transcriptions were performed with equal quantities of each total RNA as template using Quantitect Reverse Transcription Kit (TaKaRa, Japan) for real-time PCR according to the manufacturer’s protocol. qPCR was carried out according to the methods described in our previous studies (Cheng et al., 2017; Wei et al., 2018). The primer sequences of each gene used in this analysis are the same as Supplementary Table 2. A set of 10 housekeeping genes (*18S* rRNA, *β-actin, rpl7, tuba, b2m, elfa, gapdh, tbp, hprt*, and *ubce*) were selected in order to test their transcriptional stability. The relative expression of genes was calculated by 2^-ΔΔ*Ct*^ method and GeNorm was used to normalize the geometric mean of two best combination genes under different experimental conditions.

### Statistical Analysis

Results are presented as means with their standard errors (mean ± SEM). Before the statistical analysis, all data were tested for normality of distribution using the Kolmogornov–Smirnov test. Data were evaluated using one-way ANOVA, Homoscedasticity analysis and Duncan’s multiple range tests. The analysis was carried out using the SPSS 19.0 for Windows (SPSS, Chicago, IL, United States), and the minimum significant level was set at 0.05.

## Results

### Molecular Characterization

In the present study, the full-length cDNA sequences of nine SUMOylation-related genes (*sumo1, sumo2, sumo3, sae1, uba2, ubc9, pias1, senp2*, and *senp3*) were successfully obtained by RT-PCR and RACE methods from *P. fulvidraco*. Their sequences were 1239, 856, 1262, 1429, 2123, 842, 2023, 2220, and 2402 bp, respectively (Table 1). We also cloned and obtained the partial cDNA sequence of *senp1*, which was 1899 bp. We have made exhaustive efforts to clone the full-length cDNA sequence of *senp1* but met failure. The pair-wise amino acid sequence comparison of 10 sumoylation-related genes between different species were shown in Table 2. The amino acid identities of *P. fulvidraco sumo1, sumo2, sumo3, sae1, uba2, ubc9, pias1, senp1, senp2*, and *senp3* were similar to those of orthologous from other species, exhibiting 28.12–100% amino acid sequence identities among different species.

**Table 1:** The sequence information of SUMOylation-related genes from *P. fulvidraco.*

Gene	Accession no.	5′ UTR (bp)	ORF (bp)	3′ UTR (bp)	Full length (bp)	Protein (aa)
*sumo1*	MH192975	115	303	821	1239	100
*sumo2*	MH192976	21	288	547	856	95
*sumo3*	MH192977	99	285	878	1262	94
*sae1*	MH192978	93	1047	289	1429	348
*uba2*	MH192979	76	1935	112	2123	644
*ubc9*	MH192980	164	474	204	842	157
*pias1*	MH192981	13	1926	84	2023	641
*senp1*	MH192982	89	1746	64	1899	581
*senp2*	MH192983	140	1761	319	2220	586
*senp3*	MH192984	176	1620	606	2402	539


**sumo*, small ubiquitin-related modifier; *sae1*, SUMO-activating enzyme subunit 1; *uba2*, SUMO-activating enzyme subunit 2; *ubc9*, SUMO-conjugating enzyme 9; *pias1*, protein inhibitor of activated STAT; *senp*, sentrin-specific protease.*

**Table 2:** Amino acid sequence identities of SUMOylation-related genes between *P. fulvidraco* and other species (%).

Genes	*Ictalurus punctatus*	*Danio rerio*	*Bos taurus*	*Rattus norvegicus*	*Homo sapiens*
*sumo1*	97.00	91.00	82.18	82.18	82.18
*sumo2*	97.89	96.88	96.84	96.84	96.84
*sumo3*	100.00	97.87	87.50	81.82	88.35
*sae1*	93.97	87.64	69.54	66.38	68.66
*uba2*	94.14	85.52	76.73	77.04	77.35
*ubc9*	99.37	99.37	98.73	98.73	98.73
*pias1*	92.60	83.99	75.15	73.72	73.72
*senp1*	68.50	56.17	34.17	34.67	36.88
*senp2*	82.31	56.71	29.50	28.28	28.12
*senp3*	85.71	55.07	43.50	43.20	42.69


*Accession numbers as follows (the order is *Ictalurus punctatus, Danio rerio, Bos taurus, Rattus norvegicus*, and *Homo sapiens*): *sumo1* (XP_017338022.1, NP_998324.1, NP_001030535.1, NP_001009672.1, NP_003343.1); *sumo2* (NP 001187682.1, NP_001003422.1, AAI02380.1, NP_598278.1, NP 008868.3); *sumo*3 (AHH43056.1, NP_998289.1, NP_001069917.1, NP_001019466.1, NP_008867.2); *sae1* (ADO27732.1, NP_001002058.1, AAI33520.1, NP_001012063.1, NP_005491.1); *uba2* (AHH39415.1, NP_998528.1, AAI23591.1, NP_001094049.1, NP_005490.1); *ubc9* (NP_001187932.1, NP_571908.1, NP_001092842.1, NP_037182.1, NP_003336.1); *pias1* (AHH39964.1, XP_692921.3, NP_001068864.1, NP_062637.2, AAD49722.1); *senp1* (XP_017342708.1, XP_001343517.1, NP_001193805.1, XP_017450802.1, NP_001254524.1); *senp2* (XP_017336876.1, XP_684283.2, NP_001178289.1, NP_083733.1, AAH40609.1); *senp3* (XP_017327429.1, NP_001098584.1, NP_001178303.1, NP_001157043.1, AAH80658.1).*

**sumo*, small ubiquitin-related modifier; *sae1*, SUMO-activating enzyme subunit 1; *uba2*, SUMO-activating enzyme subunit 2; *ubc9*, ubiquitin-conjugating enzyme 9; *pias1*, protein inhibitor of activated STAT; *senp*, sentrin-specific protease.*

The protein sequence of *P. fulvidraco sumo1, sumo2*, and *sumo3* revealed similar domains with mammals, including the UBQ (ubiquitin-like proteins) domain, the hydrophobic surface, the Ulp1-Smt3 interaction sites, a VKTE motif and the C-terminal Gly residues (Supplementary Figure 1). The *P. fulvidraco sae1* consisted of three conserved regions, such as the E1 enzyme family domain, the ThiF domain and the dimer interaction sites (Supplementary Figure 2). The *P. fulvidraco uba2* comprised E1 enzyme family domain, the Ubiquitin/SUMO-activating enzyme ubiquitin-like domain (UAE-Ubl), and the SUMO-activating enzyme subunit 2 C-terminus (UBA2-C). The E1 enzyme domain of *P. fulvidraco uba2* included several specific hits, such as the putative ATP binding sites, putative substrate interface, the catalytic residue and the putative zinc binding sites (Supplementary Figure 3). The protein sequence of *P. fulvidraco ubc9* contained the UBCc domain, E3 interaction residues, active cysteine residue and Ub thioester intermediate interaction residues (Supplementary Figure 4). The protein sequence of *P. fulvidraco pias1* revealed all the characteristic features of the PIAS protein family, including the SAP (Saf-A/B, Acinus, and Pias) domain, the PINIT domain, a DWNN domain and the MIZ/SP-RING zinc finger. The MIZ/SP-RING zinc finger contained SP-RING finger motif and Zn binding sites (Supplementary Figure 5). As the member of SUMO-specific proteases, *senp1, senp2*, and *senp3* from *P. fulvidraco* possessed three distinct functional domains, such as the ubiquitin and ubiquitin-like protein 1 domain (Ulp1), the PLN03189 domain (protease specific for SUMO) and the C-terminal catalytic domain (Supplementary Figures 6–8).

Phylogenetic tree was performed with putative amino acid sequences, and further revealed the inferred evolutionary relationship. For *sumo, P. fulvidraco sumo2, sumo3*, and *sumo1* were separated into two clades. *sumo2* and *sumo3* were in a clade but *sumo1* clustered separately (Supplementary Figure 9). For E1 activated enzymes, *sae1* and *uba2* clustered close with *Ictalurus punctatus* but far from *Poecilia reticulata, Poecilia formosa*, and *Poecilia latipinna*. The *P. fulvidraco ubc9* was closer to teleost and more distant from insecta and crustacea, in agreement with established taxonomic relationship. *pias1* from *P. fulvidraco* clustered with *Ictalurus punctatus* but appeared far from the cluster of amphibians, aves and mammals. According to the amino acid sequences of *P. fulvidraco sae1, uba2, ubc9* and *pias1*, all teleost formed an independent cluster, while mammals formed another cluster (Supplementary Figures 10–13). Similar to *sumo, senp1, senp2*, and *senp3* were clearly divided into three clades, and *senp2* was closer to *senp1* (Supplementary Figure 14). Moreover, phylogenetic tree indicated that these amino acid sequences from *P. fulvidraco* were closer to the corresponding members of fish than those of amphibians and mammals, suggesting that the names of the genes were correct.

### Tissue Distribution of Gene Expression

**FIGURE 1:**
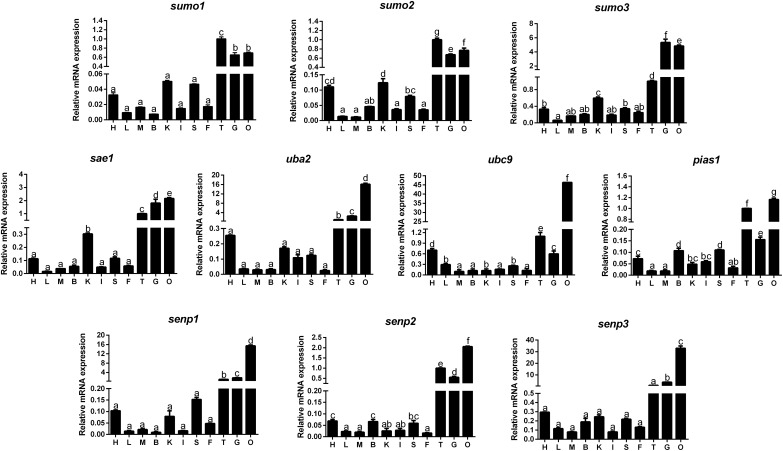
Quantitative PCR (Q-PCR) analysis for tissue distribution of gene expression across heart (H), liver (L), muscle (M), brain (B), kidney (K), intestine (I), spleen (S), mesenteric fat (F), testis (T), gill (G) and ovary (O) of *Pelteobagrus fulvidraco*. Data (mean ± SEM, *n* = 3) were expressed relative to expression of housekeeping gene [*β-actin* and *elfa* (*M* = 0.6245)]. Bars that share different letters indicate significant differences among groups (*P* < 0.05).

The mRNA levels of 10 SUMOylation-related genes were detected in all the tested tissues, but at variable levels (Figure 1). Generally speaking, these genes showed relatively high mRNA levels in ovary, gill and testis, and lowest mRNA levels in liver, muscle, intestine, and mesenteric fat.

### Transcriptional Responses of SUMOylation-Related Genes to Different Dietary Carbohydrate in Distinct Tissues

**FIGURE 2:**
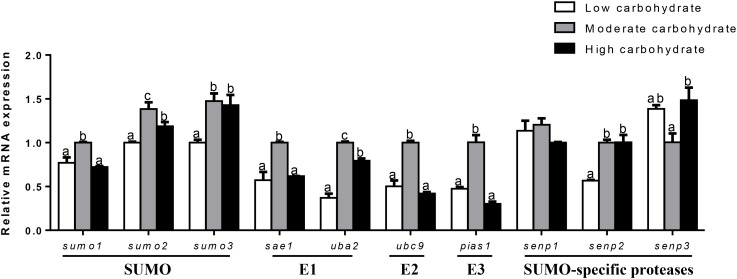
Effect of different dietary carbohydrate levels on hepatic mRNA expression of SUMOylation related genes in *P. fulvidraco*. ^a,b,c^Values are mean ± SEM, *n* = 3 (replicates of three fish) and data were expressed relative to expression of housekeeping gene [*β-actin* and *hprt* (*M* = 0.2627)]; bars that share different letters within the same gene indicate significant differences among three groups (*P* < 0.05).

The mRNA abundances of SUMOylation-related genes in liver, muscle, testis, and ovary were responsive to three dietary carbohydrate levels. In liver (Figure 2), the mRNA levels of *sumo1, sae1, ubc9*, and *pias1* were the highest for fish fed the middle carbohydrate diets and showed no significant differences between other two groups. mRNA levels of *sumo2* and *uba2* were the highest for fish fed the middle carbohydrate diet and lowest for fish fed the control. mRNA levels of *sumo3* and *senp2* were lowest for fish fed the control and showed no significant differences between other two groups. *senp3* mRNA levels were the highest fish fed the highest carbohydrate diets and showed no significant differences between other two groups. *senp1* mRNA levels showed no significant differences among three groups.

**FIGURE 3:**
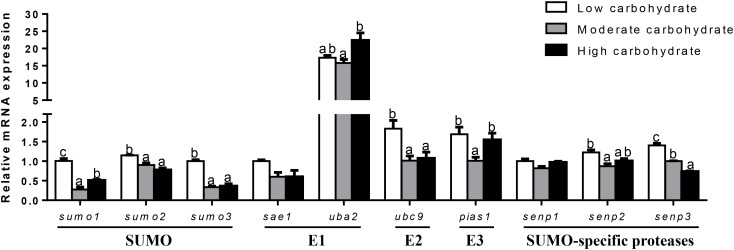
Effect of different dietary carbohydrate levels on muscle mRNA expression of SUMOylation related genes in *P. fulvidraco*. ^a,b,c^Values are mean ± SEM, *n* = 3 (replicates of three fish) and data were expressed relative to expression of housekeeping gene [*elfa* and *tbp* (*M* = 0.0484)]; bars that share different letters within the same gene indicate significant differences among groups (*P* < 0.05). The absence of different letters indicates no significant differences.

In muscle (Figure 3), *sumo1* mRNA levels were the highest for fish fed the control and lowest for fish fed middle carbohydrate diet. mRNA levels of *sumo2, sumo3, ubc9*, and *senp2* were highest for fish fed the control and showed no significant differences between other two groups. *uba2* mRNA levels were the highest for fish fed the highest carbohydrate diets and showed no significant differences between other two groups. *pias1* mRNA levels were the lowest for fish fed the middle carbohydrate diets and showed no significant differences between other two groups. *senp3* mRNA levels declined with increasing dietary carbohydrate levels. mRNA levels of *sae1* and *senp1* showed no significant differences among three groups.

**FIGURE 4:**
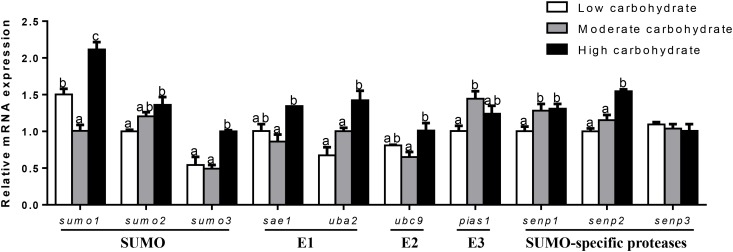
Effect of different dietary carbohydrate levels on testis mRNA expression of SUMOylation related genes in *P. fulvidraco*. ^a,b,c^Values are mean ± SEM, *n* = 3 (replicates of three fish) and data were expressed relative to expression of housekeeping gene [*rpl7* and *ubce* (*M* = 0.1046)]; bars that share different letters within the same gene indicate significant differences among groups (*P* < 0.05). The absence of different letters indicates no significant differences.

In the testis (Figure 4), *sumo1* mRNA levels were the highest for fish fed the highest carbohydrate diet and lowest for fish fed the middle carbohydrate diet. mRNA levels of *sumo2, uba2, senp1*, and *senp2* increased with increasing dietary carbohydrate levels. mRNA expression of *sumo3, sae1*, and *ubc9* was the highest for fish the highest carbohydrate diets and showed no significant differences between other two groups. *pias1* mRNA expression was the highest for fish fed the middle carbohydrate diets and showed no significant differences between other two groups. *senp3* mRNA expression showed no significant differences among three groups.

**FIGURE 5:**
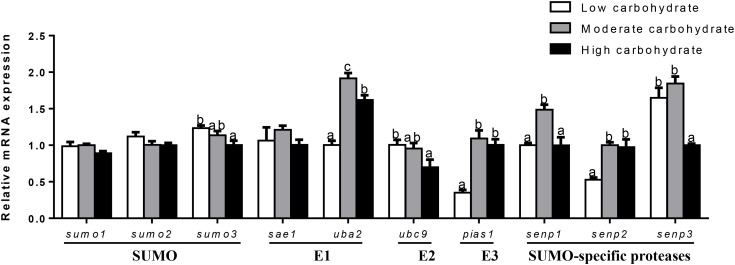
Effect of different dietary carbohydrate levels on ovarian mRNA expression of SUMOylation related genes in *P. fulvidraco*. ^a,b,c^Values are mean ± SEM, *n* = 3 (replicates of three fish) and data were expressed relative to expression of housekeeping gene [*β-actin* and *tbp* (*M* = 0.1636)]; bars that share different letters within the same gene indicate significant differences among groups (*P* < 0.05). The absence of different letters indicates no significant differences.

In ovary (Figure 5), mRNA expression of *sumo1, sumo2*, and *sae1* showed no significant differences among three groups. mRNA expression of *sumo3* and *ubc9* declined with increasing dietary carbohydrate levels. *uba2* mRNA abundance was the highest for fish fed the middle carbohydrate diets and lowest for fish the control. mRNA expression of *pias1* and *senp2* were the lowest for fish fed the control and showed no significant differences between other two groups. *senp3* mRNA expression was the lowest for fish fed highest carbohydrate diet and showed no significant differences between other two groups. *senp1* mRNA levels were the highest for fish fed the middle carbohydrate diet and showed no significant differences between other two groups.

### Effect of Glucose Incubation on Cell Viability, mRNA Expressions of Sumoylation Related Genes in Primary Hepatocytes From Yellow Catfish

**FIGURE 6:**
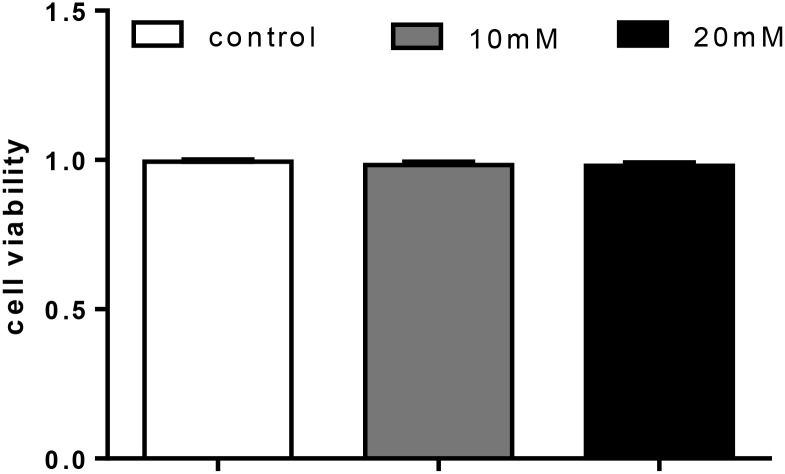
Effect of glucose [control (5.41 mmol/L), 10 mM (9.75 mmol/L), 20 mM (20.38 mmol/L)] on cell livability in primary hepatocytes from yellow catfish, and incubation lasted for 48 h. Values are mean ± SEM, *n* = 3 (replicates of three biological experiments); Bars that did not mark different letters indicate no significant differences among groups (*P* < 0.05).

Glucose treatment showed no significant effect on cell viability among the three treatments (Figure 6).

**FIGURE 7:**
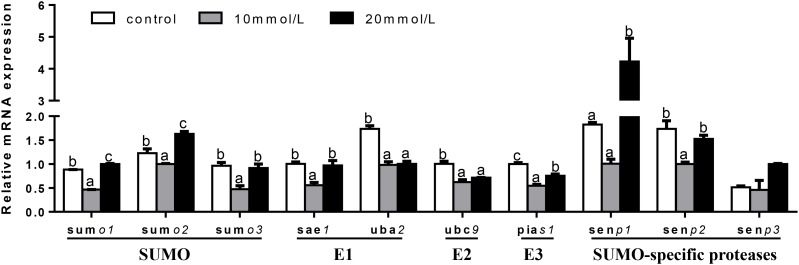
Effects of glucose (control, 10 and 20 mM) incubation on mRNA expressions of SUMOylation related genes in primary hepatocytes from yellow catfish, and incubation lasted for 12 h. ^a,b,c^Values are mean ± SEM, *n* = 3 (replicates of three biological experiments) and data were expressed relative to expression of housekeeping gene [*b2m* and *rpl7* (*M* = 0.1078)]; bars that share different letters within the same gene indicate significant differences among groups (*P* < 0.05).

At 12 h (Figure 7), mRNA expression of *senp3* showed no significant differences among three groups. *sumo1* and *sumo2* mRNA abundances were the highest for 20 mM glucose and the lowest for 10 mM glucose. mRNA expressions of *sumo3, sae1*, and *senp2* were the lowest for 10 mM glucose and showed no significant differences between other two groups. *uba2* and *ubc9* mRNA abundances were the highest for control group, *senp1* mRNA levels were the highest for 20 mM glucose and showed no significant differences between other two groups. *pias1* mRNA abundance was the highest for control group and the lowest for 10 mM glucose.

**FIGURE 8:**
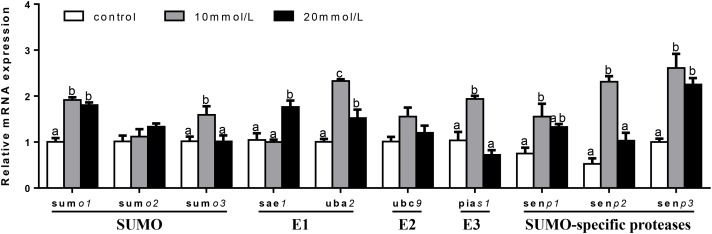
Effects of glucose (control, 10 and 20 mM) incubation on mRNA expressions of SUMOylation related genes in primary hepatocytes from yellow catfish, and incubation lasted for 24 h. ^a,b,c^Values are mean ± SEM, *n* = 3 (replicates of three biological experiments) and data were expressed relative to expression of housekeeping gene [*hprt* and *rpl7* (*M* = 0.0808)]; bars that share different letters within the same gene indicate significant differences among groups (*P* < 0.05).

At 24 h (Figure 8), mRNA expression of *sumo2* and *ubc9* showed no significant differences among three groups. *sumo1* and *senp3* mRNA abundances were the lowest for control group and showed no significant differences between other two groups. *sumo3, pias1*, and *senp2* mRNA abundances were the highest for 10 mM glucose and showed no significant differences between other two groups. *sae1* mRNA expression was the highest for 20 mM glucose and showed no significant differences between other two groups. mRNA expressions of *uba2* and *senp1* were the highest for 10 mM glucose and the lowest for control group.

**FIGURE 9:**
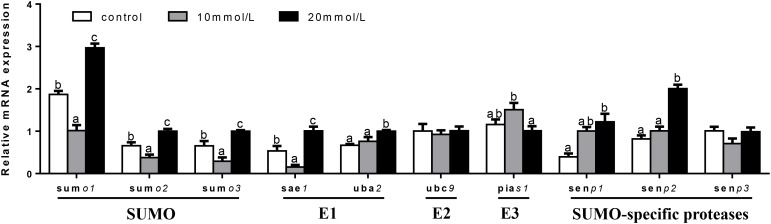
Effects of glucose (control, 10 and 20 mM) incubation on mRNA expressions of SUMOylation related genes in primary hepatocytes from yellow catfish, and incubation lasted for 48 h. ^a,b,c^Values are mean ± SEM, *n* = 3 (replicates of three biological experiments) and data were expressed relative to expression of housekeeping gene [*ubce* and *rpl7* (*M* = 0.0994)]; bars that share different letters within the same gene indicate significant differences among groups (*P* < 0.05).

At 48 h (Figure 9), mRNA expression of *ubc9* and *senp3* showed no significant differences among three groups. *sumo1, sumo2, sumo3*, and *sae1* mRNA levels were the highest for 20 mM glucose and the lowest for 10 mM glucose. *uba2* and *senp2* mRNA abundances were the highest for 20 mM glucose and showed no significant differences between other two groups. *pias1* mRNA expression was the highest for 10 mM glucose and the lowest for 20 mM glucose. mRNA levels of *senp1* increased with increasing glucose concentrations.

## Discussion

SUMOylation plays many important roles in signaling pathway, transcriptional regulation, and cell cycle progression. In the present study, for the first time, we identified the cDNA sequences of 10 SUMOylation genes, explored their mRNA tissue expression profiles and transcriptional responses to carbohydrate levels both *in vivo* and *in vitro*, which establish a basis for further investigation on their functions.

In the present study, the protein sequences of *P. fulvidraco sumo1, sumo2*, and *sumo3* revealed similar domains with mammals, indicating that the protein’s structure and function of SUMO were highly evolutionarily conserved, as reported by Yuan et al. (2010). *sumo1, sumo2*, and *sumo3* in yellow catfish had the conserved Gly–Gly motifs in their C-terminus, which are the critical region for their maturation and conjugation (Bayer et al., 1998; Xu et al., 2016). Comparing to *sumo1, sumo2/sumo3* of yellow catfish possess a VKTE motif, consistent with the SUMOylation consensus YKXE (Y represents a hydrophobic amino acid, X means any amino acid), and this consensus sequence is functional for possible polymerization (Tatham et al., 2001). In contrast, in mud crab, Dai et al. (2012) reported that SUMO1 also contained an AKPE motive site.

The present study also indicated that the *P. fulvidraco sae1* consisted of the conserved ThiF domain. The ThiF domain in *sae1* was related to the nucleotide-binding function (Dou et al., 2005). The *P. fulvidraco uba2* comprised E1 enzyme family domain, the Ubiquitin/SUMO-activating enzyme ubiquitin-like domain (UAE-Ubl), and the SUMO-activating enzyme subunit 2 C-terminus (UBA2-C). These conserved domains of *uba2* were similar to these in mammals (Schwartz and Hochstrasser, 2003; Huang et al., 2004). In the present study, the protein sequence of *P. fulvidraco ubc9* contained the UBCc domain, E3 interaction residues, active cysteine residue and Ub thioester intermediate interaction residues. Similar domains of *ubc*9 have been reported by Hu and Chen (2013). The PIAS protein family acts as an E3 ubiquitin ligase in the SUMOylation pathway. Our study indicated that the protein sequence of *P. fulvidraco pias1* revealed all the characteristic features of the PIAS protein family, including the SAP (after SAF-A/B, Acinus, and PIAS) domain, the Pro-Ile-Asn-Ile-Thr (PINIT) domain, a DWNN domain and the MIZ/SP-RING zinc finger, in agreement with other reports (Duval et al., 2003; Huang et al., 2015). A RING finger signature confers an E3-SUMO ligase function (Palvimo, 2007).

SENP1, SENP2, and SENP3 all belong to SUMO-specific proteases. As an unique crucial deSUMOylation-specific proteinase, SENPs play an important role to determine the intracellular SUMOylation level (Xia et al., 2016). In the present study, *senp1, senp2*, and *senp3* from *P. fulvidraco* possessed three distinct functional domains, such as the ubiquitin and ubiquitin-like protein 1 domain (Ulp1), the PLN03189 domain and the C-terminal catalytic domain, in agreement with other reports (Drag and Salvesen, 2008). The C-terminal catalytic domain of ubiquitin-like 1 domain contains the catalytic triad Cys-His-Asn and appears to function with sSUMOylation and deSUMOylation (Mossessova and Lima, 2000).

In this work, we examined the tissue distribution of these identified genes as a preliminary step to shed some light on their physiological roles. The present study demonstrated that the mRNA levels of 10 SUMOylation-related genes were detected in all the tested tissues, suggesting that they participated in general SUMOylation reactions occurring at basal levels in all tissues. In fish, to our best knowledge, mRNA tissue expression profiles of SUMOylation-relevant genes have been reported only in *ubc9* (Hu and Chen, 2013), *sumo1* and *sumo2* (Xu et al., 2016) and *pias1* (Wong, 2013) and these studies indicated that they were widely distributed. Moreover, our study indicated that these genes showed relatively high mRNA levels in ovary, gill and testis, and lowest mRNA levels in liver, muscle, intestine, and mesenteric fat. Similarly, several studies have demonstrated that *sumo1* gene was highly expressed in the ovary (Shen et al., 1996; Kamitani et al., 1998; Dai et al., 2012) and testis (Shen et al., 1996). Huang et al. (2015) found the higher expression level of *Pias1* in the gonad of *S. paramamosain*, and implied that *pias1* may exert an important function in the maintenance of spermatogenesis. High expression of these genes in the ovary implies that the SUMOylation and deSUMOylation were highly active in the regulation of gonad development (Hu and Chen, 2013). In mouse, Ihara et al. (2008) also reported that *Ubc9* had an important role in the development of oocytes. High mRNA expression of *sumo1* and *sumo2* in gills also had been reported in grouper (Xu et al., 2016). The gills were primary sites where fish encounter environmental stresses. Therefore, we can reasonably infer that the high expression level of these genes in the gills might be a sensor for environmental stress in fish that stimulates the SUMOylation and deSUMOylation responses.

In mammals, several studies indicated that SUMOylation modification of proteins were influenced by carbohydrate (or glucose) levels (Huang et al., 2013; Jung et al., 2016), but no such studies were conducted in fish. For the first time, our study explored the effects of dietary carbohydrate levels on mRNA expressions of the 10 genes in four tissues in fish, and detected the effect of glucose incubation on mRNA expressions of SUMOylation related genes in primary hepatocytes from yellow catfish. Our study indicated that dietary carbohydrate levels differentially influenced their mRNA expression in a dose- and tissue-dependent manner, and glucose incubation differentially influence their mRNA expression in dose- and time-dependent ways. At present, we did not know the exact reason and further mechanism for carbohydrate (glucose)-induced changes of their mRNA expression levels. However, since these genes and proteins mediated the regulation of many transcriptional factors and signaling pathways related to nutrient metabolism (Sentis et al., 2005; Chung et al., 2011; Koo et al., 2015), it is understandable that the carbohydrate-induced changes of these gene expression will in turn influence the utilization and metabolism of carbohydrate in fish. Similarly, Huang et al. (2013) reported that high glucose increased the expression of *sumo1* and *sumo2/3* in glomerular mesangial cells of the rats. Jung et al. (2016) pointed out that different stimulations were required for inducing different SENPs according to cell types, and concluded that chronic high glucose was responsible for inducing *Senp2* expression in insulin-producing cells.

## Conclusion

In summary, for the first time, we characterized the cDNA sequences of 10 SUMOylation related genes from yellow catfish, determined their transcriptional responses to different levels of dietary carbohydrate, and detected the effect of different levels glucose on mRNA expressions of SUMOylation related genes in primary hepatocytes, which would contribute to our understanding of the molecular basis of SUMOylation and the potential mechanisms of SUMOylation modifications in influencing carbohydrate metabolism in fish. Further studies should be directed to analyze the functional roles of these identified genes and proteins, and clarify the mechanism of dietary carbohydrate levels influencing their transcriptional responses.

## Author Contributions

S-BY and ZL designed the experiments. S-BY conducted the experiments, with the help of X-YT, D-GZ, and JC. S-BY, ZL, and X-YT analyzed the data. S-BY drafted the manuscript. ZL revised the manuscript. All the authors read and approved the manuscript.

## Conflict of Interest Statement

The authors declare that the research was conducted in the absence of any commercial or financial relationships that could be construed as a potential conflict of interest.

## Footnotes

1 http://www.ncbi.nlm.nih.gov/Structure/cdd/wrpsb.cgi

2 http://smart.embl-heidelberg.de/

3 http://guidance.tau.ac.il/

## Abbreviations

DMSO: dimethyl sulfoxide
FBS: fetal bovine serum
MTT: 3-(4,5-dimethylthiazol-2-yl)-2,5-diphenyltetrazoliumbromide
ORF: open reading fame
PBS: phosphate buffered saline
PIAS1: protein inhibitor of activated STAT1
RACE: rapid-amplification of cDNA ends
RT-PCR: reverse transcription-PCR
Sae1: SUMO-activating enzyme subunit 1
SAP: Saf-A/B, Acinus and Pias
SEM: standard error of mean
SENP: sentrin-specific protease
Smt3: yeast ubiquitin-like protein
SUMO: small ubiquitin-related modifier
UAE-Ubl: ubiquitin/SUMO-activating enzyme ubiquitin-like protease
Uba2: SUMO-activating enzyme subunit 2
UBA2-C: SUMO-activating enzyme subunit 2 C-terminus
Ubc9: ubiquitin-conjugating enzyme 9
UBCc: ubiquitin-conjugating enzyme E2 catalytic
UBQ: ubiquitin and ubiquitin-like protein
Ulp1: ubiquitin-like protease 1
