# Supplementary material for: Identification of 10 SUMOylation-Related Genes From Yellow Catfish Pelteobagrus fulvidraco, and Their Transcriptional Responses to Carbohydrate Addition in vivo and in vitro
Source: Front Physiol. 2018 Nov 8;9:1544. doi: 10.3389/fphys.2018.01544 (PMC6235910; doi:10.3389/fphys.2018.01544)
Supplement: Supplementary file 1 [file Data_Sheet_1.PDF]

Supplementary Table 1. Nucleotide sequences of the primers used for the cDNA cloning from *P. fulvidraco*.

Primers	Sequences (5'-3')
**Primers for 3'-RACE PCR**	
3' *sumo1*-outer	CAGATAACCAGACTCCCAAAG
3' *sumo1*-inner	TCAGACATCTGCACCGCTAT
3' *sumo2*-outer	ATCAACCTGAAGGTGGCG
3' *sumo2*-inner	CCTACTGTGAACGACAGGGAC
3' *sumo3*-outer	GATGCTGGGATCGAGAGGAGCGA
3' *sumo3*-inner	ATGCCCAGCCAGCCTTAT
3' *sae1*-outer	CAAGTAGAAAGCAAGCCAGAT
3' *sae1*-inner	GGAGGCCAAACGCAATC
3' *uba2*-outer	CACAGCGTACCATGACAGC
3' *uba2*-inner	GCAGATAGAGGATGGCAAGG
3' *ubc9*-outer	CTCCCAGTTCATCAGGTTCA
3' *ubc9*-inner	TTTGGGAGGAGACGAGGG
3' *pias1*-outer	CTGCCCATATCGCTTCTG
3' *pias1*-inner	ACGAACTCATAAAGCCCACC
3' *senp1*-outer	GCAGCCAGTCATCATTCTTC
3' *senp1*-inner	GGCAGTGACTGTGGGATGTT
3' *senp2*-outer	AGCAAAGTCTGTGAGGTCGTA
3' *senp2*-inner	CGTAAAGTGATGATCTGGGAGA
3' *senp3*-outer	TTGCTCCTTATTTGATGGTGA
3' *senp3*-inner	TCCGCAGATTTGCTACGAG
3' RACE Outer	TACCGTCGTTCCACTAGTGATTT
3' RACE Inner	CGCGGATCCTCCACTAGTGATTTCACTATAGG
**Primers for 5'-RACE PCR**	
5' *sumo1*-outer	CTGGGTGAAGCACAAAGG
5' *sumo1*-inner	TTGGGAGTCTGGTTATCTGC
5' *sumo2*-outer	AAGCGGATCTGCCTCATT
5' *sumo2*-outer	GTTTGCTGAGCGGCGTAT
5' *sumo3*-outer	GTCCTCGTCTTCCATCTCC
5' *sumo3*-inner	GTCCGTCAAACCTAAACCTAATC

5' *sae1*-outer	TCTGACCATCTGCGTCCA
5' *sae1*-inner	CACCCAGTCCTCTGAGTCCA
5' *uba2*-outer	CTCAGCTCCATCGCCTTT
5' *uba2*-inner	ATGCAGTGGATGGGTTCG
5' *ubc9*-outer	ACAGCATCCGCAGTTTGA
5' *ubc9*-inner	CCAAGCCTTTCGTTCCTG
5' *pias1*-outer	TGGGCTTCTTCTCATTCATT
5' *pias1*-inner	GTCTGCTGGGACGCTTTG
5' *senp1*-outer	CCTCAGTCAACTCTGGGAAT
5' *senp1*-inner	AAAGGCTGGATAAACCGAGA
5' *senp2*-outer	TCATAATGGTGCGGCTTG
5' *senp2*-inner	AACTGCGACACCGTTCGA
5' *senp3*-outer	TTGCTCCTTATTTGATGGTGA
5' *senp3*-inner	TCCGCAGATTTGCTACGAG
5' RACE Outer	CTAATACGACTCACTATAGGGC
5' RACE Inner	CTAATACGACTCACTATAGGGCA AGCAGTGGTATCAACGCAGAGT

Abbreviations: *sumo*, small ubiquitin-related modifier; *sae1*, SUMO-activating enzyme subunit 1; *uba2*, SUMO-activating enzyme subunit 2; *ubc9*, ubiquitin-conjugating enzyme 9; *pias1*, protein inhibitor of activated STAT; *senp*, sentrin-specific protease.

Supplementary Table 2. Primers used for real-time quantitative PCR analysis

Genes	Forward primer (5'-3')	Reverse primer (5'-3')	Size	Accession no.
sumo1	TGGCCAGGACAACA GTGAAA	CTAGTCGTTCCAGT AGCCGC	226	MH192975
sumo2	CGAGAAGCCCAAGG AAGGAG	TGTCCGTCTCGTTA ATGGGC	206	MH192976
sumo3	CGCCGCTCAGCAAGT TAATG	CCGCCCGTTTGTTG TTGAAA	166	MH192977
sae1	TCTGACCTGGGCTTG GAGTA	TAATCTGGTGTCGT GCGCTT	224	MH192978
uba2	CTGGTCAACGTTGTG CACTG	CATGGTGCTGGAG GAAGGTT	225	MH192979
ubc9	TGATGAACTGGGAGT GTGCC	GCCTCCAGTCTTTG TCCTCC	201	MH192980
pias1	CTCCTGCTGAACCGT TTCCT	GAGCTGTAGATGT CCGAGCC	185	MH192981

senp1	CGCCTCATCATCACC AGGAA	TTCTTAGTCCAGCG CCGTAC	200	MH192982
senp2	TCCTCATCCCTTTGC ACCTG	GCTGGTCACCGTCC ACTTTA	194	MH192983
senp3	AGAGCTGGAAAACA TGGCGA	TGCCGTGATACCCG TTCAAA	201	MH192984
18s rRNA	AGCTCGTAGTTGGAT CTCGG	CGGGTATTCAGGCG AGTTTG	196	KP938527
β-actin	GGACTCTGGTGATGG TGTGA	CTGTAGCCTCTCTC GGTCAG	138	EU161066
rpl7	GGCAAATGTACAGG AGCGAG	GCCTTGTTGAGCTT GACGAA	199	KP938522
tuba	TCAAAGCTGGAGTTC TCGGT	AATGGCCTCGTTAT CCACCA	135	KP938526
b2m	GCTGATCTGCCATGT GAGTG	TGTCTGACACTGCA GCTGTA	186	KP938520
elfa	GTCTGGAGATGCTGC CATTG	AGCCTTCTTCTCAA CGCTCT	151	KU886307
gapdh	TTTCAGCGAGAGAG ACCCAG	ATGACTCTCTTGGC ACCTCC	132	KP938521
tbp	AGCAAAGAGTGAGG AGCAGT	ACTGCTGATGGGT GAGAACA	170	KP938525
hprt	ATGCTTCTGACCTGG AACGT	TTGCGGTTCAGTGC TTTGAT	181	KP938523
ubce	TCAAGAAGAGCCAG TGGAGG	TAGGGGTAGTCGAT GGGGAA	150	KP938524

Abbreviations: *sumo*, small ubiquitin-related modifier; *sae1*, SUMO-activating enzyme subunit 1; *uba2*, SUMO-activating enzyme subunit 2; *ubc9*, ubiquitin-conjugating enzyme 9; *pias1*, protein inhibitor of activated STAT; *senp*, sentrin-specific protease; *rpl7*, ribosomal protein L7; *tuba*, tubulin alpha chain; *b2m*, beta-2-microglobulin; *elfa*, translation elongation factor; *gapdh*, Glyceraldehyde-3-phosphate dehydrogenase; *tbp*, TATA-box-binding protein; *hprt*, hypoxanthine-guanine phosphoribosyltransferase; *ubce*, ubiquitin-conjugating enzyme.

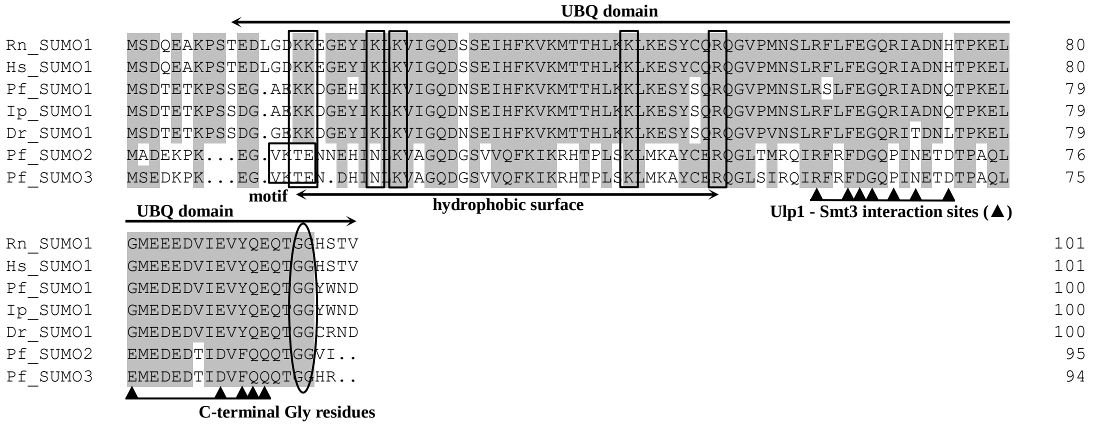

Supplementary Fig. 1. Multiple amino acid sequences alignment of SUMO1, SUMO2 and SUMO3 from *P. fulvidraco* and other species. Accession numbers as follows (the order is *Pelteobagrus fulvidraco* (Pf), *Ictalurus punctatus* (Ip), *Danio rerio* (Dr), *Rattus norvegicus* (Rn) and *Homo sapiens* (Hs)): MH192975, MH192976, MH192977, XP_017338022.1, NP_998324.1, NP_001009672.1, NP_003343.1). Arrow above the sequences represents the ubiquitin-like proteins (UBQ) domain. The hydrophobic surface and “VKXE” motif are boxed. AA with oval frame suggests the conserved C-terminal Gly residues. Residues below symbols (▲) are identified as Ulp1 - Smt3 interaction sites.

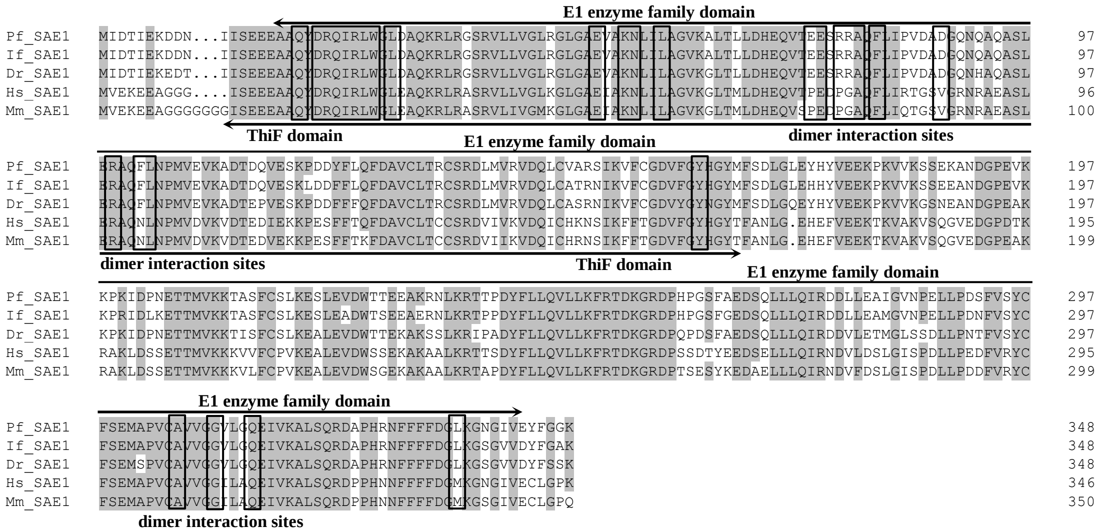

Supplementary Fig. 2. Multiple amino acid sequences alignment of SAE1 from *P. fulvidraco* and other species. Accession numbers as follows (the order is *Pelteobagrus fulvidraco* (Pf), *Ictalurus punctatus* (Ip), *Danio rerio* (Dr), *Rattus norvegicus* (Rn) and *Homo sapiens* (Hs)): MH192978, ADO27732.1, NP_001002058.1, NP_001012063.1, NP_005491.1). Arrow above the sequences represents the E1 enzyme family domain, and the arrow below the sequences represent ThiF domain. The dimer interaction sites are boxed.

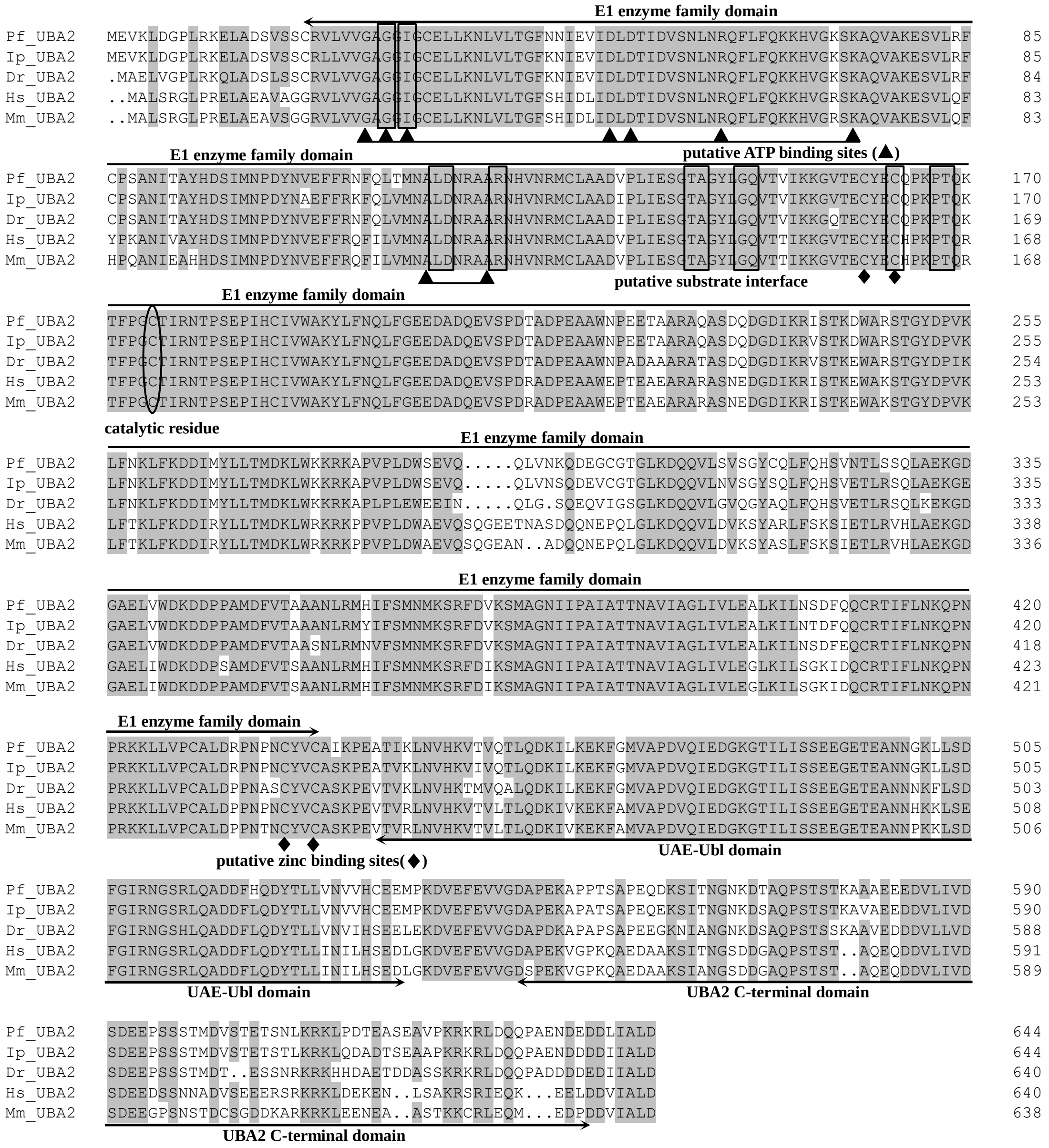

Supplementary Fig. 3. Multiple amino acid sequences alignment of UBA2 from *P. fulvidraco* and other species. Accession numbers as follows (the order is *Pelteobagrus fulvidraco* (Pf), *Ictalurus punctatus* (Ip), *Danio rerio* (Dr), *Rattus norvegicus* (Rn) and *Homo sapiens* (Hs)): MH192979, AHH39415.1, NP_998528.1, NP_001094049.1, NP_005490.1). Arrow above the sequences represents the E1 enzyme family domain, and the arrow below the sequences represents the Ubiquitin/SUMO-activating enzyme ubiquitin-like (UAE-Ubl) domain, and the SUMO-activating enzyme subunit 2 C-terminus (UBA2-C) domain. The putative substrate are boxed. AA with oval frame suggests the catalytic residues. Residues below symbols (▲) are identified as putative ATP binding sites and residues below symbols (◆) are identified as putative Zn binding sites.

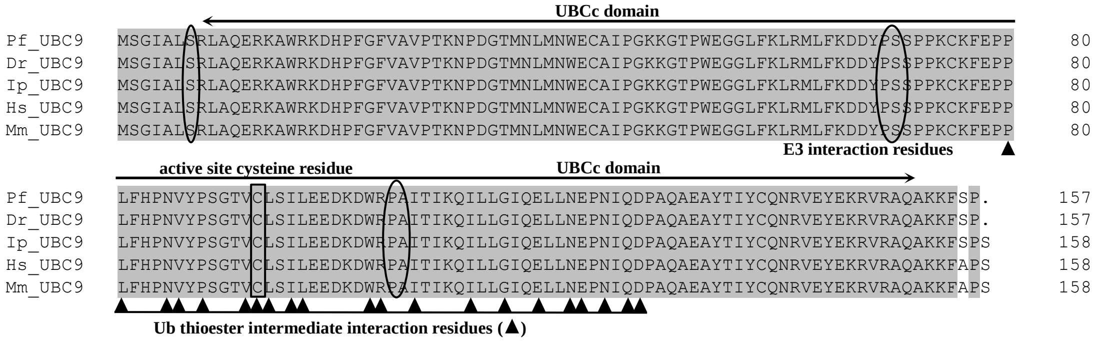

Supplementary Fig. 4. Multiple amino acid sequences alignment of UBC9 from *P. fulvidraco* and other species. Accession numbers as follows (the order is *Pelteobagrus fulvidraco* (Pf), *Ictalurus punctatus* (Ip), *Danio rerio* (Dr), *Rattus norvegicus* (Rn) and *Homo sapiens* (Hs)): MH192980, NP_001187932.1, NP_571908.1, NP_037182.1, NP_003336.1). Arrow above the sequences represents the UBCc domain. The active site cysteine residue is boxed. AA with oval frame suggests the E3 interaction residues. Residues below symbols (▲) are identified as Ub thioester intermediate interaction residues.

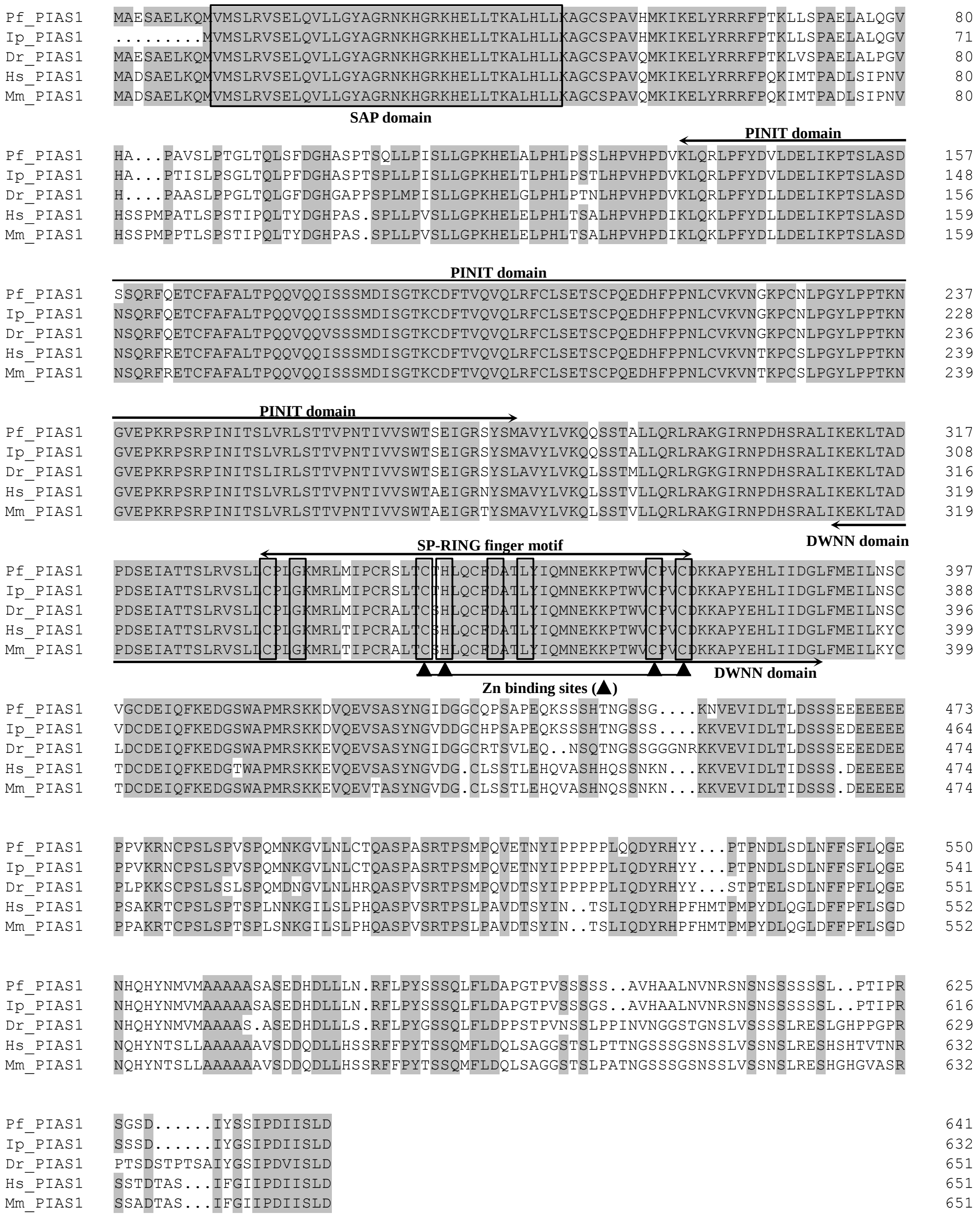

Supplementary Fig. 5. Multiple amino acid sequences alignment of PIAS1 from *P. fulvidraco* and other species. Accession numbers as follows (the order is *Pelteobagrus fulvidraco* (Pf), *Ictalurus punctatus* (Ip), *Danio rerio* (Dr), *Rattus norvegicus* (Rn) and *Homo sapiens* (Hs)): MH192981, AHH39964.1, XP_692921.3, NP_001100299.2, AAD49722.1). Arrow above the sequences represents the PINIT domain and arrow below the sequences represents DWNN domain. The SAP (after SAF-A/B, Acinus and PIAS) domain and MIZ/SP-RING zinc finger are boxed. Residues below symbols (▲) are identified as Zn binding sites.

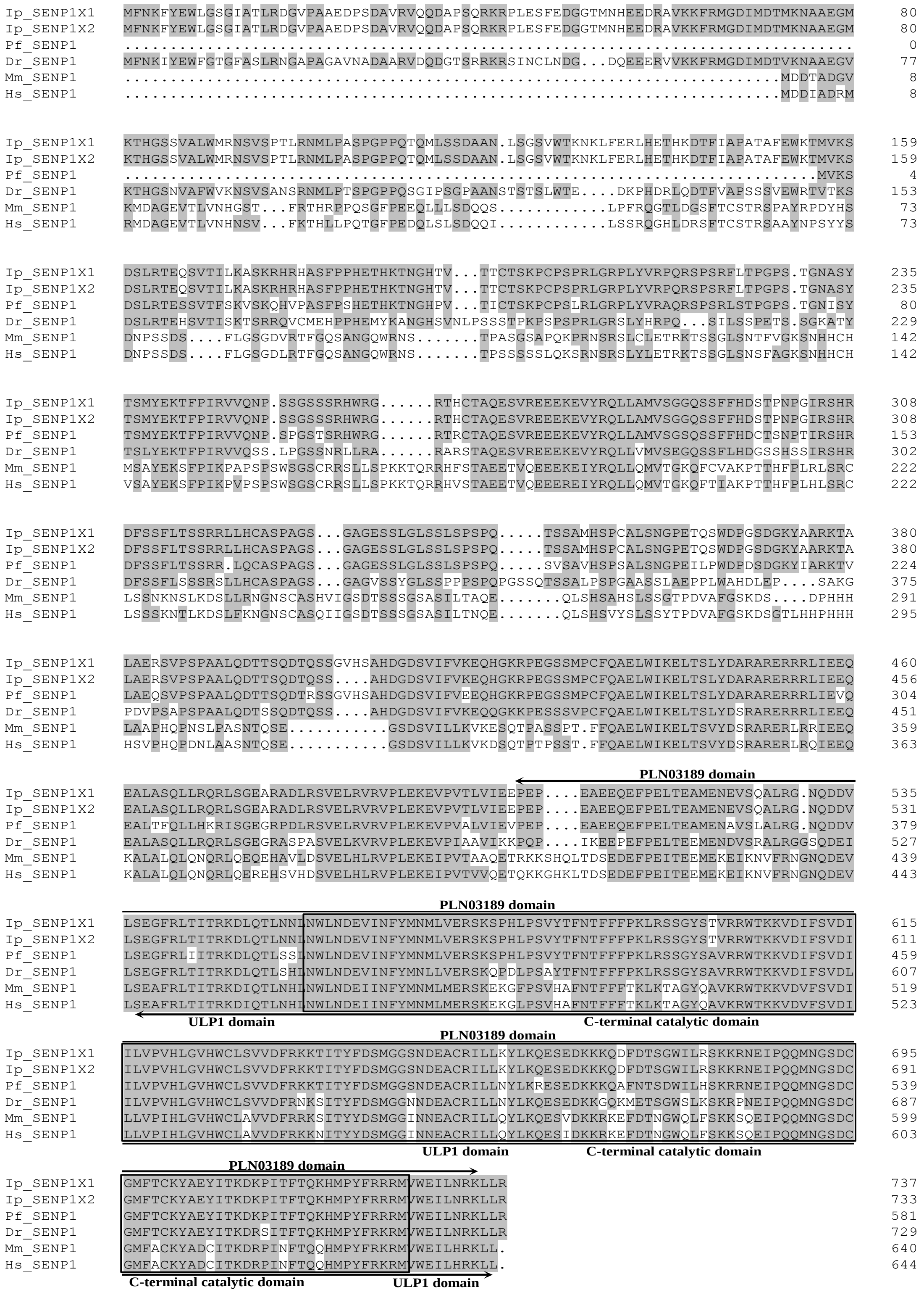

Supplementary Fig. 6. Multiple amino acid sequences alignment of SENP1 from *P. fulvidraco* and other species. Accession numbers as follows (the order is *Pelteobagrus fulvidraco* (Pf), *Ictalurus punctatus* (Ip), *Danio rerio* (Dr), *Rattus norvegicus* (Rn) and *Homo sapiens* (Hs)): MH192982, XP_017342708.1, XP_017342709.1, XP_001343517.1, XP_017450802.1, NP_001254524.1). Arrow above the sequences represents the PLN03189 domain (protease specific for SUMO) and arrow below the

sequences represents the ubiquitin and ubiquitin-like protease 1 (ULP1) domain. The C-terminal catalytic domain is boxed.

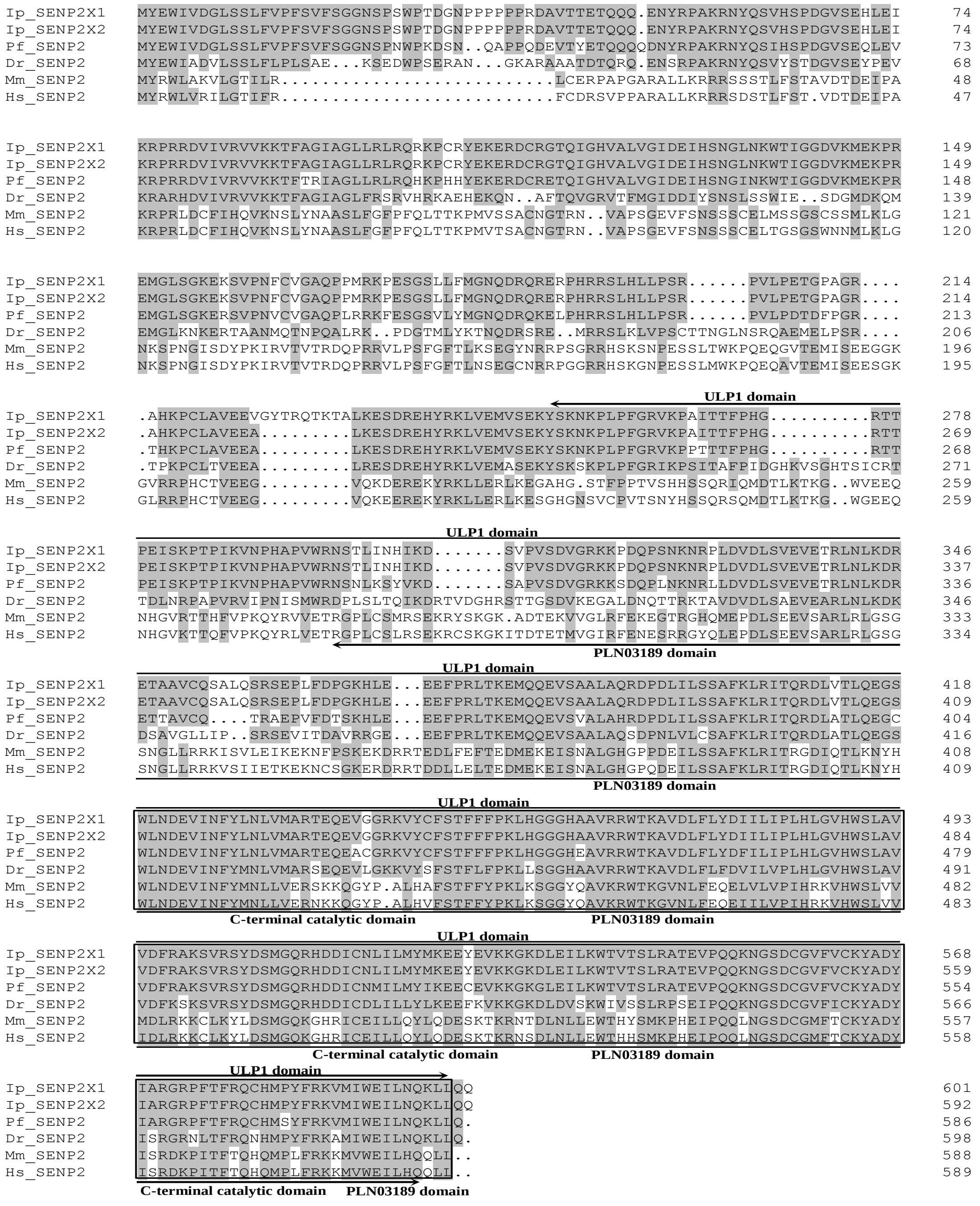

Supplementary Fig. 7. Multiple amino acid sequences alignment of SENP2 from *P. fulvidraco* and other species. Accession numbers as follows (the order is *Pelteobagrus fulvidraco* (Pf), *Ictalurus punctatus* (Ip), *Danio rerio* (Dr), *Rattus norvegicus* (Rn) and *Homo sapiens* (Hs)): MH192983, XP_017336876.1, XP_017336877.11, XP_684283.2, NP_076479.1, AAH40609.1). Arrow above the sequences represents the ubiquitin and ubiquitin-like protease 1 (ULP1) domain and arrow below the sequences represents the PLN03189 domain (protease specific for SUMO). The C-terminal catalytic domain is boxed.

```
Pf_SENP3     MRDSGASLAPNRWQGELSLTVSHEGSGGG..MAGGHLLGPDSP.PNAQSAMDFKVGHKE...RGWTGEYIKTDEM     69
Ip_SENP3     MRDSGASLAPNRWQGELSLTVSHEGSGGG..MAGGHLLGPESP.PNAQSAMDFKVGHKE...RGWTGEYIETDEM     69
Dr_SENP3b    ................................MGGGLMDPNSP.PNATSPIHLKLSNRE...RVWSEDYIEPVEL     39
Dr_SENP3a    MRDSGSSLAQTHWSNELTLTAGQEGAGGGGAIGGGHLMNPAMA.QHAPSPAHFKLGRREEDVRMWGADYTVPEVE     74
Rn_SENP3     MKETIQGTG..SWGPEPPGPGT..TYSNP...RRERLRWPLPPKPRLKSGGGFGPDPGS.GTTVPTRRLPGPRPS     67
Hs_SENP3     MKETIQGTG..SWGPEPPGPGIPPAYSSP...RRERLRWPPPPKPRLKSGGGFGPDPGS.GTTVPARRLPVPRPS     69

Pf_SENP3     DEEDGFDEDDD..EEELEEDQLDEGEE.ISSECAPVEEEEGVVDWNDPHDFLP..QMAHYYQNFSPSNKEQDKPE    139
Ip_SENP3     DEEDGFDEEEE..EEELEEDQLDEGEE.ISSECAPVEEEEDMGDWHDPHKSLP..QMAHYYQNFSPSNKEQDKPE    139
Dr_SENP3b    DEEAGFDENEDDMEEDEEEDEEERDEEDIDSECAPWEEKDDLEDWQMPFESQPHVMLANTYQQQVTLTEGEAKSQ    114
Dr_SENP3a    DEE....EEEE..EEEEDDVEMESGEDLEDQGIFHLQDQGDDKDWGTPEHSQV...............AEMDVRV    128
Rn_SENP3     FDASASEEEEEEEEED....EEEVAAWRLPPRWGQLGTSQRPRALRPSHRKTCSQRRRRAMRAFQMLLYSKSTSL    138
Hs_SENP3     FDASASEEEEEEEEEEDEDEEEEVAAWRLPPRWSQLGTSQRPRPSRPTHRKTCSQRRRRAMRAFRMLLYSKSTSL    144

Pf_SENP3     VRDVPER.PLWARLNGLRQQRLKRWR.RLRASTRLRFRLAQSWKTWRQRAQWVGTLGYRRARR.WRQYNLYPSRQ    211
Ip_SENP3     ARDDPER.PLWARLHGLRQQRLKRWR.RLRASTRLRFRLAQNWKTWRQRAQWVGTLGYRRARR.WRQYNLYPSRQ    211
Dr_SENP3b    ARDFSEN.PFQMRLHGLRQQRLNRWR.RLRSSARLRNRLVQNWKTWRQQAQWVGTLGYRRARR.WRQYSLYASK.    185
Dr_SENP3a    TDDFTHC.MAQRRPRLLKVRRLARLPNRGRRYLSIRFRLAQHWRTWRQRAKWAGTMGYRRVRR.CHRRRYFGSQH    201
Rn_SENP3     TFHWKLWGRHRGRRRNLAHPKNHLSPQEGGATPQVPSPCCRFDSPRGLPPPRLGLLGALMAEDGMRGSPPVPSGP    213
Hs_SENP3     TFHWKLWGRHRGRRRGLAHPKNHLSPQQGGATPQVPSPCCRFDSPRGPPPPRLGLLGALMAEDGVRGSPPVPSGP    219

Pf_SENP3     .RRDLGSNISRVTVQ...TANES.ETEAPFSDLNGYHGIPQSS.........SAVSVGNSERQDFPLVS.KQRVE    271
Ip_SENP3     .RRDPGLPNSRAAAQ...TANES.DTAS.FSDLNGYHSISQSS.........SAVSVGNNERQGFSSVS.MQRVE    270
Dr_SENP3b    .KREKGCDTKGSTLRGLVTTSER.ETENQFSAYNGYGDSQSVS.........SLSGVREYLSMPMKPT..IQRIE    247
Dr_SENP3a    FQRPPGRSLLNGDPEGRLQGTEISDSCSTNGALKGRDGLHGAV.........LGEREGRASPTNVQSQREKASLQ    267
Rn_SENP3     PMEEDGLRWTPKSPLDPDSGLLSCTLPNGFGGLSGPEGERSLAPPDASILISNVCSIGDHVAQELFQSSDLGTAE    288
Hs_SENP3     PMEEDGLRWTPKSPLDPDSGLLSCTLPNGFGGQSGPEGERSLAPPDASILISNVCSIGDHVAQELFQGSDLGMAE    294

                                                               PLN03189 domain
                                                    <-------------------------
Pf_SENP3     LA............LTKEHVSCVQGILDESLHKYGSLIPIHTDDIVEQLQEIFNESFSQAYRKAEVHQIIQSYQR    334
Ip_SENP3     MA............LTKEHVSCVQGILDESLHKYGSLIPIHTDDIVEQLQEIFSESFSQAYRKAEVHQIIQSYQR    333
Dr_SENP3b    MT............LTEEHKAYVHGLIEDDLRKYGSLIPVHADDIAEQLQSIFNEDFSQTHRKMAVQHLIQSYQR    310
Dr_SENP3a    NQ............LSSEHISCVQGILDEFLQQYGSLIPIHVDEVVEKLQEIFTESFSSPHRKIMVQHLMQSYQR    330
Rn_SENP3     EADRPGEKAGQHSPLREEHVTCVQSILDEFLQTYGSLIPLSTDEVVEKLEDIFQQEFSTPSRKSLVLQLIQSYQR    363
Hs_SENP3     EAERPGEKAGQHSPLREEHVTCVQSILDEFLQTYGSLIPLSTDEVVEKLEDIFQQEFSTPSRKGLVLQLIQSYQR    369

                                    PLN03189 domain
             ---------------------------------------------------------------------------
Pf_SENP3     SPGTSMVRGFSVNYKRHVLTMDDLSTLYGQNWLNDQVMNMYGDLVMDSVPEKVHFFNSFFYDKLRTKGYEGVKRW    409
Ip_SENP3     SPGTSMVRGFSVNYKRHVLTMDDLSTLYGQNWLNDQVMNMYGDLVMDSVPDKVHFFNSFFYDKLRTKGYEGVKRW    408
Dr_SENP3b    SSGTAMVKGFRVNYKRHVLTMDDLSTLYGQNWLNDQVMNMYGDLVMDSVPEKVHFFNSFFYDKLRTKGYDGVKRW    385
Dr_SENP3a    MSGSAMMRGFRVNYKRHVLVMDDLTTLYGQNWLNDQVMNMYGDLVMDAAPEKVHFFNSFFYDKLRTKGYEGVKRW    405
Rn_SENP3     MPGNAMVRGFRVSYKRHVLTMDDLGTLYGQNWLNDQVMNMYGDLVMDTVPEKVHFFNSFFYDKLRTKGYDGVKRW    438
Hs_SENP3     MPGNAMVRGFRVAYKRHVLTMDDLGTLYGQNWLNDQVMNMYGDLVMDTVPEKVHFFNSFFYDKLRTKGYDGVKRW    444
                                <--------------------------------------------------
                               ULP1 domain                C-terminal catalytic domain
                                    PLN03189 domain
             ---------------------------------------------------------------------------
Pf_SENP3     TKNVDIFSKDLLLIPIHLEVHWSLVSVDIKQRFITYFDSQRTLNRRCPKHIFKYLQAKAIIKQQRDFLTGWRGFF    484
Ip_SENP3     TKNVDIFSKDLLLIPIHLEVHWSLVSVDIKQRYITYFDSQRTLNRRCPKHIFKYLQAEAIIKQQRDFLTGWRGFF    483
Dr_SENP3b    TKNVDIFQKDLLLIPIHLEVHWSLVSVDIKRRSITYFDSQRTLNRRCPKHIFKYLQAEAMIKEKRDFLTGWKGFF    460
Dr_SENP3a    TKNVDIFQKKFLLIPIHLEVHWSLVCVNVPQRSVTYFDSQRTLNRRCPKHIAKYLQAEAIKREQKDFYTGWKGFF    480
Rn_SENP3     TKNVDIFNKELLLIPIHLEVHWSLVSVDVRRRTITYFDSQRTLNRRCPKHIAKYLQAEAVKKDRLDFHQGWKGYF    513
Hs_SENP3     TKNVDIFNKELLLIPIHLEVHWSLISVDVRRRTITYFDSQRTLNRRCPKHIAKYLQAEAVKKDRLDFHQGWKGYF    519
              ---------------------------------------------------------------------------
                                    ULP1 domain           C-terminal catalytic domain
                      PLN03189 domain
             ------------------------------------------>
Pf_SENP3     KMNVGRQNNDSDCGAFVLQYCKCLALGQPFSFSQQDMPRLRRIMYKELCHCKLSL                        539
Ip_SENP3     KMNVGRQNNDSDCGAFVLQYCKCLSLGQPFSFSQQDMPKLRRLMYKELCHCKLSL                        538
Dr_SENP3b    KMNVGRQNNDSDCGAFVLQYCKCLALGQPFSFSQQDMPKLRRMMYKELCHCKLSL                        515
Dr_SENP3a    KMNVARQNNDSDCGAFVLQYCKCLALEQPFSFGQQDMPKLRRQMYKELCHCKLSL                        535
Rn_SENP3     KMNVARQNNDSDCGAFVLQYCKHLALSQPFSFTQQDMPKLRRQIYKELCHCKLTV                        568
Hs_SENP3     KMNVARQNNDSDCGAFVLQYCKHLALSQPFSFTQQDMPKLRRQIYKELCHCKLTV                        574
             --------------------------------------------------->
                     ULP1 domain          C-terminal catalytic domain
```

Supplementary Fig. 8. Multiple amino acid sequences alignment of SENP3 from *P. fulvidraco* and other species. Accession numbers as follows (the order is *Pelteobagrus fulvidraco* (Pf), *Ictalurus punctatus* (Ip), *Danio rerio* (Dr), *Rattus norvegicus* (Rn) and *Homo sapiens* (Hs)): MH192984, XP_017327429.1, NP_001098584.1, NP_001077311.1, NP_001013134.2, AAH80658.1). Arrow above the sequences represents the PLN03189 domain (protease specific for SUMO) and arrow below the sequences represents the ubiquitin and ubiquitin-like protease 1 (ULP1) domain. The C-terminal catalytic domain is boxed.

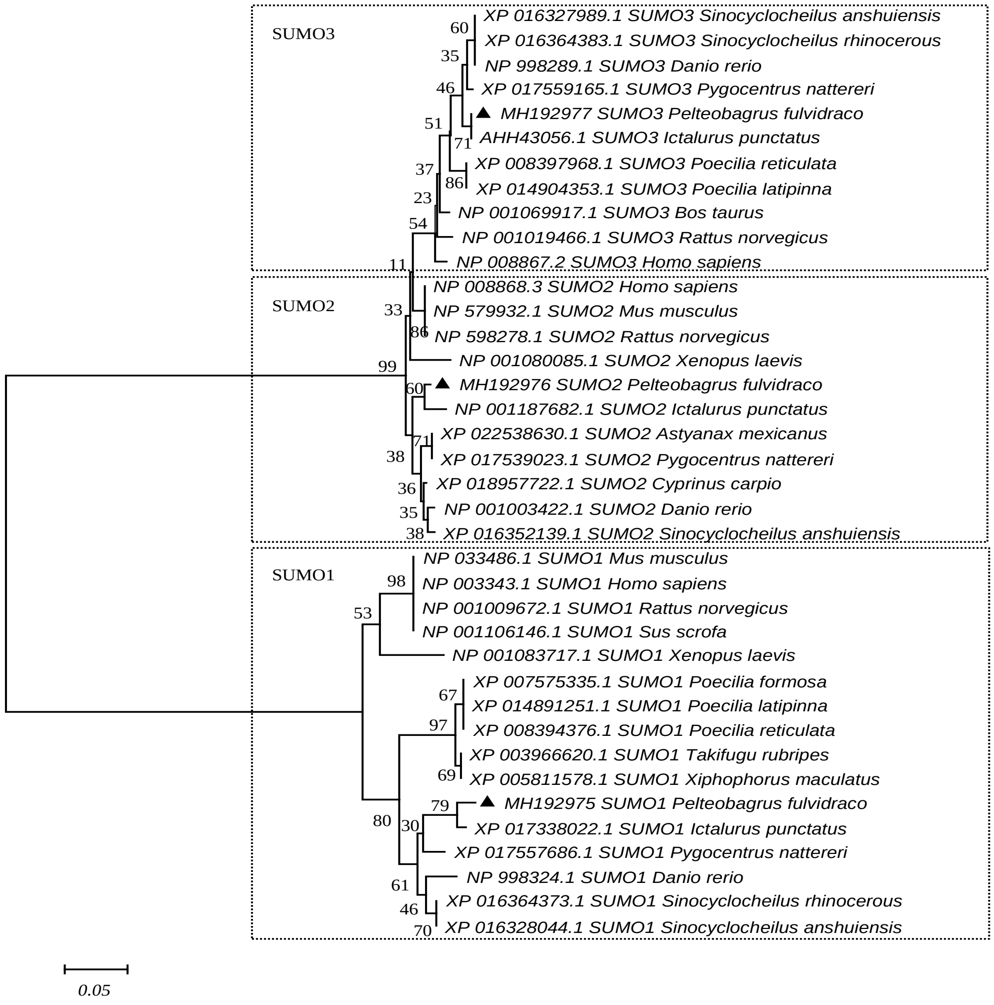

Supplementary Fig. 9. Phylogenetic tree based on the protein sequences of SUMO1, SUMO2 and SUMO3 from *P. fulvidraco* and other vertebrate species using the neighbor-joining (NJ) method in MEGA 6.0 (Tamura et al., 2013) based on the JTT+G model (Jones et al., 1992). Branch support values represent a percentage of 1000 bootstrap replicates.

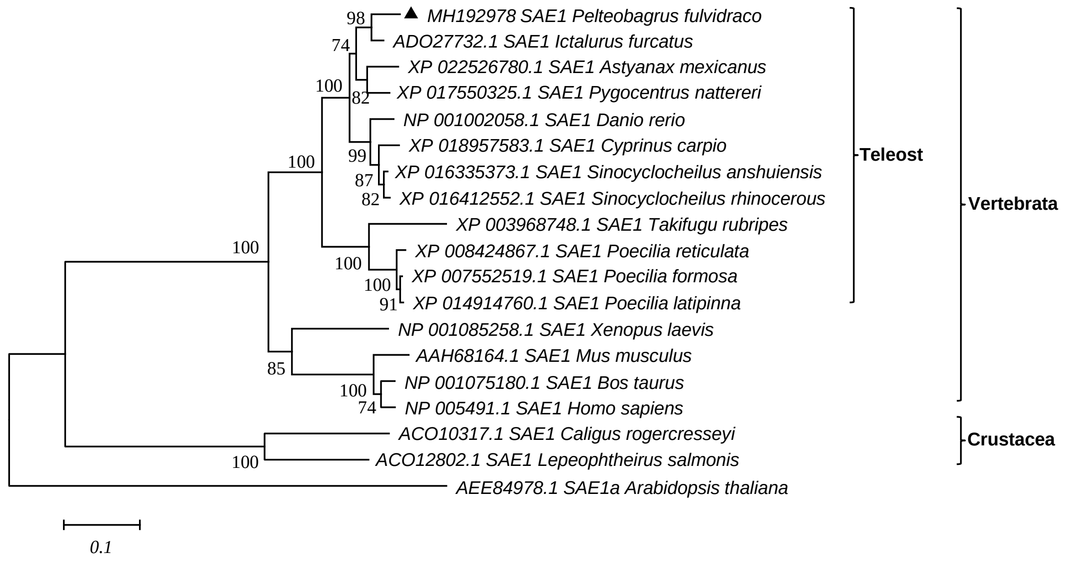

Supplementary Fig. 10. Phylogenetic tree based on the protein sequences of SAE1 from *P. fulvidraco* and other vertebrate species using the neighbor-joining (NJ) method in MEGA 6.0 (Tamura et al., 2013) based on the JTT+G model (Jones et al., 1992). Branch support values represent a percentage of 1000 bootstrap replicates.

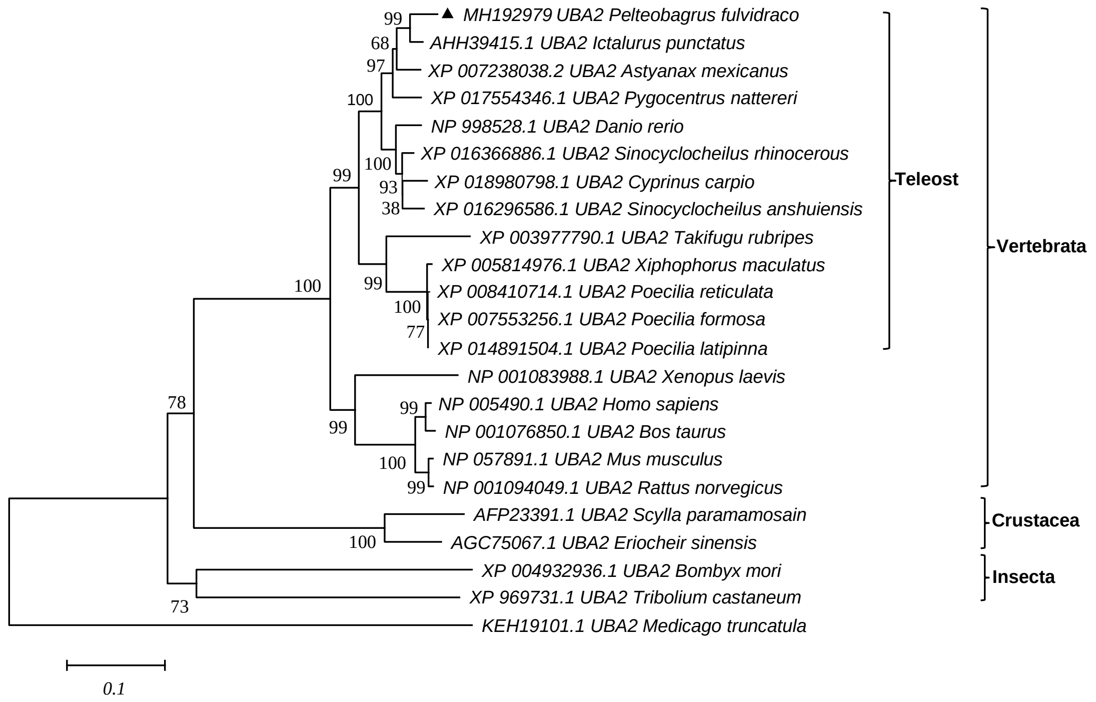

Supplementary Fig. 11. Phylogenetic tree based on the protein sequences of UBA2 from *P. fulvidraco* and other vertebrate species using the neighbor-joining (NJ) method in MEGA 6.0 (Tamura et al., 2013) based on the JTT+G model (Jones et al.,

1992). Branch support values represent a percentage of 1000 bootstrap replicates.

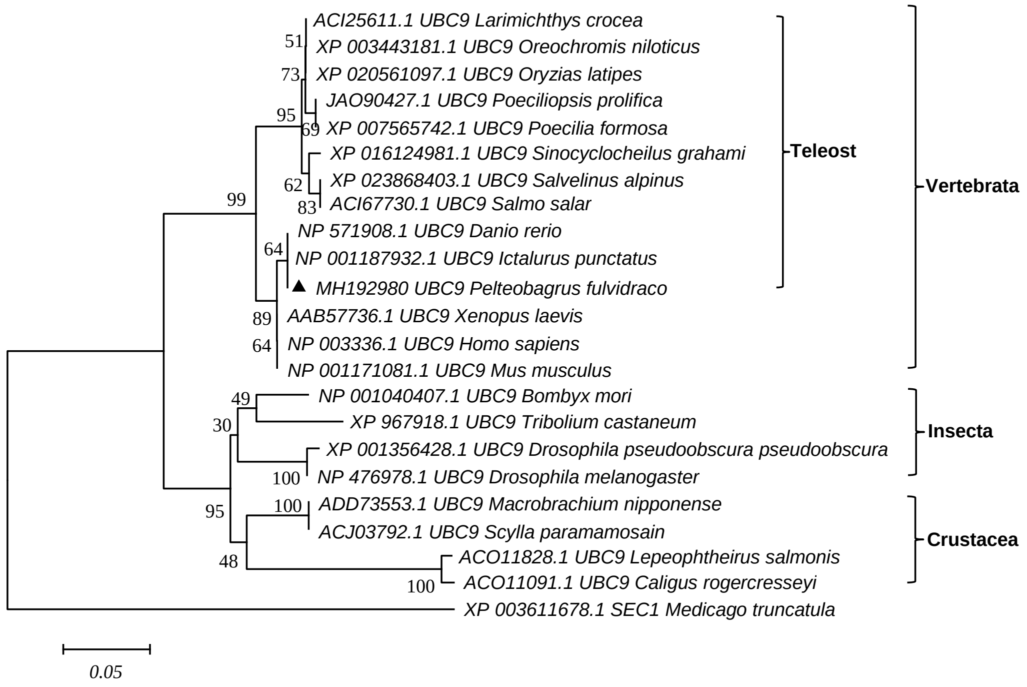

Supplementary Fig. 12. Phylogenetic tree based on the protein sequences of UBC9 from *P. fulvidraco* and other vertebrate species using the neighbor-joining (NJ) method in MEGA 6.0 (Tamura et al., 2013) based on the JTT+G model (Jones et al., 1992). Branch support values represent a percentage of 1000 bootstrap replicates.

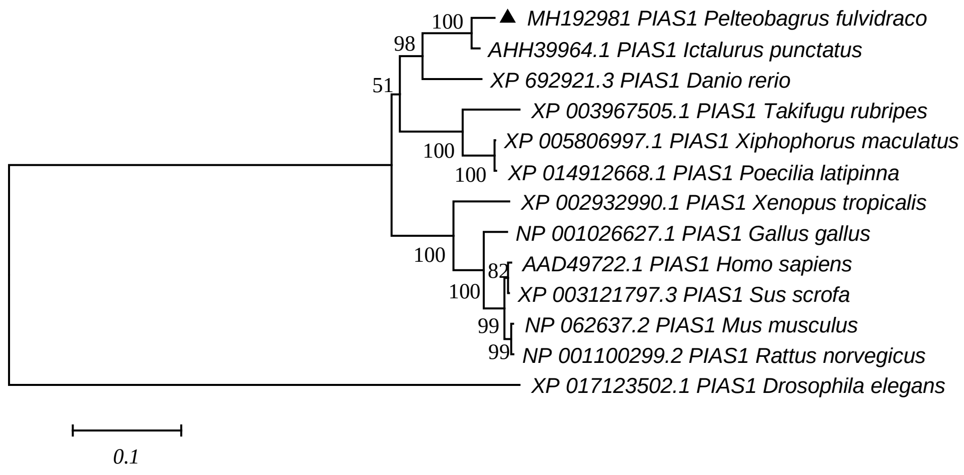

Supplementary Fig. 13. Phylogenetic tree based on the protein sequences of PIAS1 from *P. fulvidraco* and other vertebrate species using the neighbor-joining (NJ) method in MEGA 6.0 (Tamura et al., 2013) based on the JTT+G model (Jones et al., 1992). Branch support values represent a percentage of 1000 bootstrap replicates.

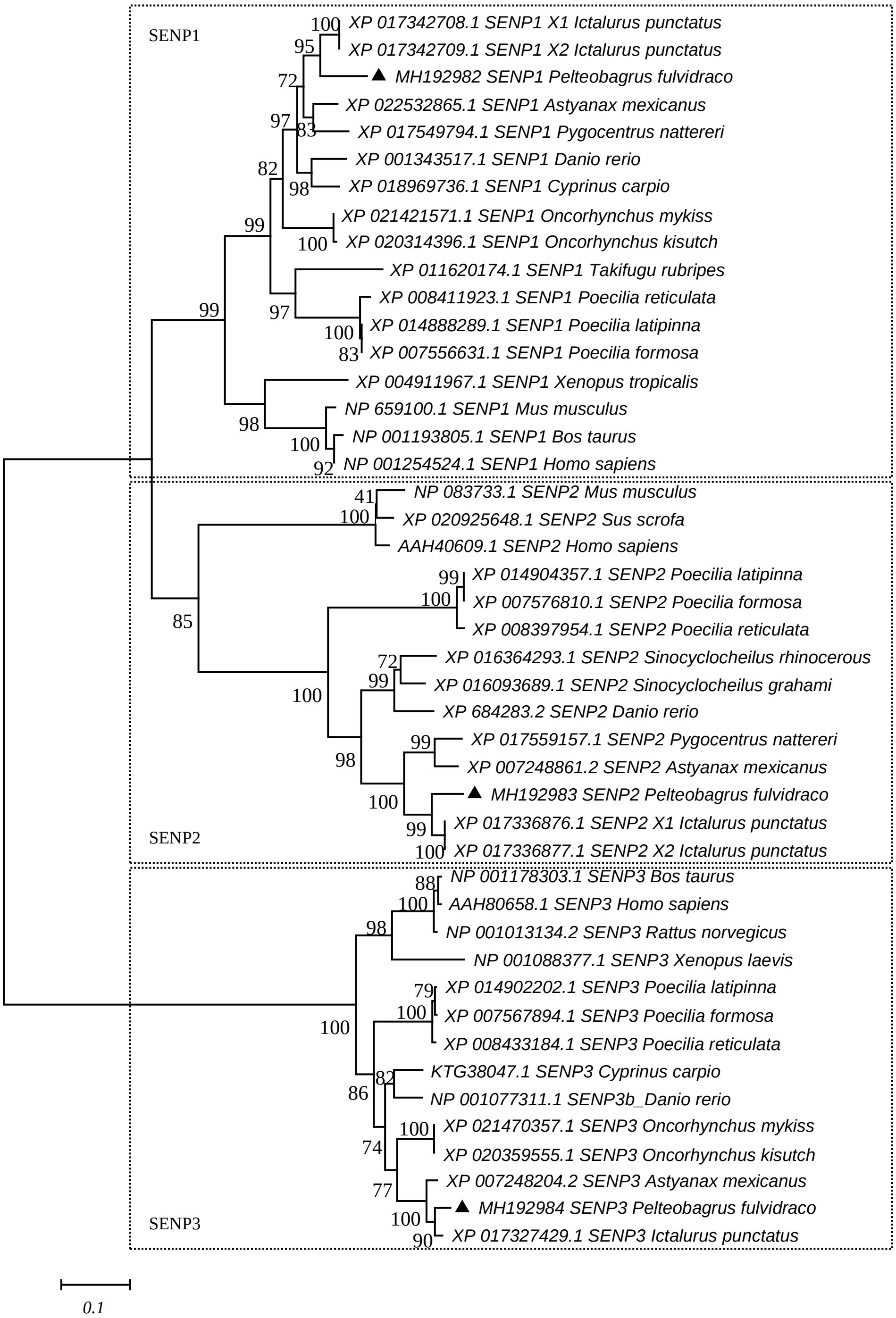

Supplementary Fig. 14. Phylogenetic tree based on the protein sequences of SENP1, SENP2 and SENP3 from *P. fulvidraco* and other vertebrate species using the neighbor-joining (NJ) method in MEGA 6.0 (Tamura et al., 2013) based on the JTT+G model (Jones et al., 1992). Branch support values represent a percentage of 1000 bootstrap replicates.
